# There’s an App for That? Highlighting the Difficulty in Finding Clinically Relevant Smartphone Applications

**DOI:** 10.5811/westjem.2015.12.28781

**Published:** 2016-03-02

**Authors:** Warren Wiechmann, Daniel Kwan, Andrew Bokarius, Shannon L. Toohey

**Affiliations:** * University of California, Irvine, Department of Emergency Medicine, Irvine, California; † UCSF-Fresno, Department of Emergency Medicine, Fresno, California; ‡ University of Chicago, Department of Emergency Medicine, Chicago, Illinois

## Abstract

**Introduction:**

The use of personal mobile devices in the medical field has grown quickly, and a large proportion of physicians use their mobile devices as an immediate resource for clinical decision-making, prescription information and other medical information. The iTunes App Store (Apple, Inc.) contains approximately 20,000 apps in its “Medical” category, providing a robust repository of resources for clinicians; however, this represents only 2% of the entire App Store. The App Store does not have strict criteria for identifying content specific to practicing physicians, making the identification of clinically relevant content difficult. The objective of this study is to quantify the characteristics of existing medical applications in the iTunes App Store that could be used by emergency physicians, residents, or medical students.

**Methods:**

We found applications related to emergency medicine (EM) by searching the iTunes App Store for 21 terms representing core content areas of EM, such as “emergency medicine,” “critical care,” “orthopedics,” and “procedures.” Two physicians independently reviewed descriptions of these applications in the App Store and categorized each as the following: Clinically Relevant, Book/Published Source, Non-English, Study Tools, or Not Relevant. A third physician reviewer resolved disagreements about categorization. Descriptive statistics were calculated.

**Results:**

We found a total of 7,699 apps from the 21 search terms, of which 17.8% were clinical, 9.6% were based on a book or published source, 1.6% were non-English, 0.7% were clinically relevant patient education resources, and 4.8% were study tools. Most significantly, 64.9% were considered not relevant to medical professionals. Clinically relevant apps make up approximately 6.9% of the App Store’s “Medical” Category and 0.1% of the overall App Store.

**Conclusion:**

Clinically relevant apps represent only a small percentage (6.9%) of the total App volume within the Medical section of the App Store. Without a structured search-and-evaluation strategy, it may be difficult for the casual user to identify this potentially useful content. Given the increasing adoption of devices in healthcare, national EM associations should consider curating these resources for their members.

## INTRODUCTION

As the adoption rates for personal mobile devices such as smartphones and tablets in the consumer space continue to rise,^1^ we are witnessing similar adoption trends amongst healthcare providers.^2^,^3^ It is estimated that 82–85% of physicians in the U.S. own a smartphone,^3^,^4^ representing an increase of 64% over only a few years ago.^4^ The computing power of these devices, despite their small size, and their constant connectivity to the Internet is contributing to their increased usage as clinical decision-making tools, quick-reference tools, and sources of medical education content. Much of the aforementioned functionality of these devices lies in their applications, or “apps” with approximately 30–50% of medical professionals using apps in clinical care environments. Given the fast-paced nature of emergency medicine (EM), rapid access to medical data contained in the apps could be particularly useful to the practicing emergency physician.

Smartphone applications can be found online in two locations – the iTunes App Store, which features apps for Apple products such as the iPhone, iPod, iPad, and iPad mini, and the Google Play Store featuring apps for the Android operating system. Each of these two stores offers a “Medical” category; however, upon a cursory review of the content it is evident that the category covers a wide range of topics including general health and wellness information; there is no categorization of apps for healthcare professionals. Physicians and medical students have anecdotally noted difficulty in identifying clinical relevant apps and this has led to the popularity of medical-app review websites. One of the most widely recognized review websites is iMedicalApps; this website uses a team of medical student and physician reviewers to curate and review clinically relevant apps across a variety of medical specialties.^5^ Apple, Inc. later published an “Apps for Healthcare Professionals” category on its App Store, although there was some critique about the breadth of apps, review criteria for identifying these selected apps, and frequency of updates.

The ability to locate apps clinically relevant to EM is seemingly more difficult than other specialties, such as dermatology or ophthalmology, since the scope of our practice also covers the acute management of diseases and conditions found in every medical specialty. While many websites contain listings of “top apps” in EM, there is not a commonly accepted or definitive collection of apps to our knowledge.

In the literature, prior studies have described app uses by percent of use of different types of apps,^3^ while other studies have discussed the need for a broader discussion across accrediting bodies to ensure ability to use high quality evidence-based apps.^6^ However, our literature review showed no prior studies that attempted to categorize clinical and non-clinical applications and quantify the number of apps available. The objective of our study was to quantify and categorize the characteristics of existing medical applications in the iTunes App Store that could be used by physicians, residents and medical students.

## METHODS

While medical apps can be found in the iTunes and Android app stores, we chose to only review apps from the iTunes Store as data suggests that a larger percentage of healthcare providers prefer to use Apple devices over other competing devices.^3^

Data collection was conducted in April 2014 by two independent reviewers: ST and DK, both EM residents. Using the iTunes application for Windows/Mac Operating Systems, the reviewers conducted a series of Boolean searches using 21 search terms. The search terms used were the following: “Anesthesia,” Critical Care,” “Dermatology,” “Emergency,” “Emergency Medicine,” “Geriatrics,” “Gynecology,” “ICU,” “Intensive Care,” “Medicine,” “Neurology,” “Obstetrics,” “Orthopedics,” “Pediatrics,” “Pharmacology,” “Procedures,” “Psychiatry,” “Radiology,” “Surgery,” “Toxicology” and “Ultrasound.” These terms were chosen to represent the main content themes of EM training.^7^ Terms such as “Pediatrics” and “Orthopedics” were used with the intention of finding specialty-specific content possibly relevant to EM that may not have been represented in an EM- specific app. Searches were conducted in the iTunes App to ensure that search results for both iPad and iPhone would be included in the analysis.

Each app listed in the search results was categorized as Clinically Relevant, Book/Published Source, Non-English, Study Tools/Reference, or Not Clinically Relevant based on review of the app’s information page and associated sample images (Table 1). Reviewers were instructed only to review the information page and not download the app, as this preliminary review mimicked app downloading behaviors described by an informal focus group of medical students and residents.^8^ A third reviewer (AB) audited any discrepancies in categorization. We then calculated descriptive statistics.

## RESULTS

At the time of data collection in Spring 2013, the iTunes App Store contained approximately one million apps, with an estimated 20,000 apps comprising the “Medical” Category (2% of the total app volume).^9^

We found a total of 7,699 apps from the 21 search terms, of which 1,372 (17.8% were clinical, 738 (9.6%) were based on a book or published source, 126 (1.6%) were non-English, 55 (0.7%) were clinically relevant patient-education resources, and 372 (4.8%) were study tools. We considered 4,994 (64.9%) not relevant to medical professionals (Figure). Clinically relevant apps make up approximately 6.9% of the App Store’s “Medical” category and 0.1% of the overall App Store. Two reviewers did the initial review of these apps, and disagreed 0.7% of the time, at which point a third reviewer settled the disagreement.

Due to the methods used to search for apps there is some overlap in the results, which likely caused a false elevation of the numbers. As a result, the true numbers are actually even lower and further highlight the difficulty of finding quality content.

## DISCUSSION

Clinically relevant apps represent only a small percentage (6.9%) of the apps within the “Medical” category” and an even smaller percentage (0.1%) of the apps in the entire iTunes App Store. Even with a structured search-and-evaluation strategy, it is evident that healthcare providers will have difficulty in identifying clinically relevant material.

While guides and curated lists of “top apps” exist on the Internet, many of these resources cite the same content,^10^–^13^ which raises the question: Are these the most relevant clinical applications or are they preferentially selected by reviewers without evaluating the full App Store content? If the latter statement is a more accurate reflection of these curated lists, it may propagate a situation where healthcare providers are not exposed to newer and potentially useful clinical apps.

These results also prompt a discussion about corporate collaboration with the medical profession and its responsibility in identifying healthcare-specific resources within their content collections. Both Apple, Inc and Google, Inc have engaged in collaborations with healthcare institutions^14^–^19^ and as stated earlier, Apple has created its own curated list of Apps for Healthcare Professionals. Despite these efforts, our data suggest that there are opportunities for further collaboration, such as redefining the “Medical” categories to represent “true medical content” and create a broader “Health/Wellness” category for consumer use. Clinically relevant categorization of apps can happen without support from companies like Apple and Google; however, the process is labor intensive and their cooperation would likely yield higher and more consistent results.

Until there is a more useful “Medical” app category, there are several great resources that review medical Apps and provide recommendations, such as iMedicalApps.com. Some of the authors’ most used and favorite Apps are listed in Table 2.

## LIMITATIONS

New apps are being added all the time. Our study is cross-sectional and limited to what was published in the iTunes App Store at the time the terms were searched and app list collected. We attempted to control for this by using screen-captures of the search results, although at the time of publication, we recognize that the makeup of the “Medical” category will have changed.

The iTunes App Store catalog is proprietary information, so it is not possible to obtain a complete list and true denominator of all apps in the “Medical” category. As such, we were only able to find apps using our chosen search terms and ultimately only looked at 7699 of approximately 20,000 apps in the medical section of the app store. However, we assume that if the applications do not contain any of the search terms of interest then they are unlikely to be medically applicable.

Additionally, there is some subjectivity in categorization of the apps. Selecting non-relevant apps was generally clear-cut, while determining what was “clinical” vs. “study tools” was disagreed upon 0.7% of the time. Lastly, we only looked at descriptions and titles to categorize the apps. We did not download each app or attempt to measure the quality of the apps reviewed. Lastly, with the increase in Android and Windows devices, a similar analysis of these apps may be useful.

## CONCLUSION

Given the increasing adoption of devices in healthcare, national EM associations should consider curating these resources for their members. Additionally, further studies evaluating the actual quality and evidence-based nature of useful medical apps is essential to the safe use of these apps in the clinical practice of EM, where rapid access to medical information is useful.

## Figures and Tables

**Figure:**
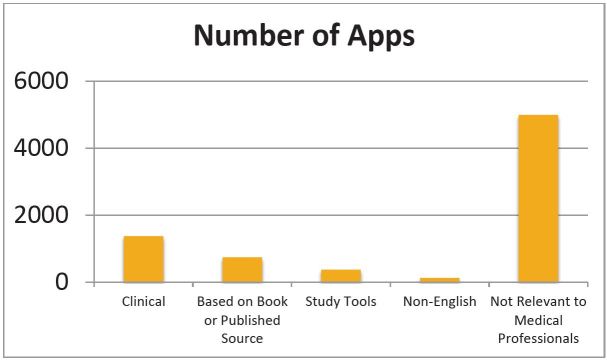
Number of applications divided into categories

**Table 1:** Definition of the variables used for categorization of iTune medical apps.

Variable	Definition	Example
Clinically relevant	App content could potentially influence or guide a clinical decision	Epocrates
Book/published source	App that served as a digital accompaniment to an existing published textbook or medical journal	*Academic Emergency Medicine* journal app
Not English	App that does not contain English	Mobile medicine medical
Study tools/reference	App that contained useful medical information, but was not intended for clinical decision-making, such as board review questions and flashcard apps	PEERVII
Not clinically relevant	App that contained no medical information, such as games or medical apps not related to healthcare professionals	1800-Contacts App

**Table 2:** Author-recommended applications.

Type	Apps
Comprehensive Emergency Medicine Apps	PEPID Emergency Medicine Suite, PalmEM, 5min EM Consult, WikiEM, palmEM, ERres
Calculators	MedCalc, NeuroToolKit
Pharmacy apps	Micromedex, Epocrates, EMRA Antibiotics Guide, EMRA Peds Meds, PressorDex
Pediatric apps	palmPEDi, Pedi Stat
Dermatology apps	VisualDx
Ultrasound	One Minute Ultrasound, SonoSupport, Pocket Atlas of Emergency Ultrasound
Toxicology apps	PEPID Elements, ACEP Toxicology Antidote App
Other apps	Eye Chart, Touch Surgery, UCSF Neuro Exam Tutor, UCSF MSK Exam Tutor
